# Effect of Yoga Intervention on Problem Behavior and Motor Coordination in Children with Autism

**DOI:** 10.3390/bs14020116

**Published:** 2024-02-04

**Authors:** Xingda Ju, Huanhuan Liu, Jing Xu, Bo Hu, Yunlei Jin, Chang Lu

**Affiliations:** 1 School of Psychology, Northeast Normal University, Changchun 130000, China; juxd513@nenu.edu.cn (X.J.); huanhuanliu211@163.com (H.L.); hub897@nenu.edu.cn (B.H.); 2 Jilin Provincial Key Laboratory of Cognitive Neuroscience and Brain Development, Changchun 130000, China; 3 School of Life Sciences, Northeast Normal University, Changchun 130000, China; xuj391@nenu.edu.cn; 4 Children’s Hospital of Changchun, Changchun 130000, China; jinyl001@outlook.com

**Keywords:** autism, yoga intervention, problem behavior, motor coordination

## Abstract

Children with autism exhibit more pronounced symptoms of both problem behaviors and motor coordination difficulties. Yoga, recognized as an effective intervention modality, can be valuable after assessing its efficacy in addressing problem behaviors and motor coordination challenges, ultimately contributing to symptom alleviation in autism. The randomized controlled trial (RCT) was used to divide 17 children with autism into an intervention group (*n* = 9) and a control group (*n* = 8). The intervention group participated in an 8-week yoga intervention training (three sessions/week, 45–50 min/session), and the control group did not participate in yoga training but only in daily program activities. Pre-test, mid-test, post-test, and after delayed test, teachers assessed the effect of yoga intervention on problem behaviors of children with autism through the Aberrant Behavior Checklist (ABC) and the effect of yoga intervention on motor coordination through the Movement Assessment Battery for Children—Second Edition (MABC2). Results show that the yoga intervention is effective in reducing problem behaviors and improving motor coordination in children with autism. Yoga intervention significantly reduces irritability and social withdrawal in children with autism. Yoga intervention had the most significant improvement in ball skills and static and dynamic balance.

## 1. Introduction

Autism Spectrum Disorder (ASD) is a developmental neurological disorder characterized primarily by an impairment or even loss of individual social functioning [1]. It manifests as reduced interest in interacting with others and social difficulties, along with narrow interests and repetitive stereotypical behaviors. Children with autism exhibit pronounced characteristics of problem behaviors and motor coordination difficulties. To address the reduction of problem behaviors and enhance motor coordination abilities in children with autism, the effectiveness of yoga intervention in addressing both aspects is explored.

### 1.1. Problem Behaviors in Autism and Alleviation Methods

When the desires of children with autism are not comprehended by parents or teachers, and cannot be fulfilled, the children often exhibit problem behaviors such as emotional outbursts, aggression, and self-injury. In individuals with autism, problem behaviors such as self-injury, hostility, stereotypic behavior, and disruptive behavior are commonly observed [2]. Some problem behaviors exhibited by individuals with autism can be linked to challenges in the social domain [3]. Parents of children with autism at risk for problem behaviors experience elevated parenting stress [4]. Therefore, focusing on the improvement of problem behaviors in children with autism not only helps to reduce the severity of their social impairment, but also helps to reduce the parenting pressure on families of children with autism.

Currently, the major approaches to reducing problem behaviors include medication and behavioral interventions [2,5]. No consensus exists regarding the use of pharmacologic treatments for autism. Despite numerous clinical observations, only a limited number of controlled studies have validated the efficacy and safety of these treatments [6]. Medication is ineffective for core symptoms of ASD [7]. Numerous medication treatments are still pending confirmation of their safety and effectiveness [8]. Interventions have emerged as a more dependable and efficacious approach. Currently, multimedia social storytelling and positive behavioral interventions are the predominant strategies for reducing problem behaviors in children with autism. The multimedia social storytelling intervention, implemented through home–school collaboration, reduced the frequency of problem behaviors in children with autism [9]. Individual-wide positive behavior support reduces problem behaviors in children with autism in inclusive settings [10]. Recent research indicates that yoga intervention significantly reduces irritability and social withdrawal associated with problem behaviors in children with autism [11]. Yoga can be used as an alternative therapy to reduce the severity of symptoms in children with autism [12].

### 1.2. Motor Coordination in Autism and Improvement Modalities

Stereotypic behavior is one of the core symptoms of children with autism [13]. The interaction of these factors, such as social interactions, sensory hypersensitivity, communication deficits, and motor incoordination, creates a complex context in which individuals with autism exhibit stereotypic behaviors, with motor incoordination acting as an important prediction of stereotypic behaviors [14]. Motor-related factors have been studied as precursors to autism [15,16,17]. For example, some researchers have used deep learning latent variable models to identify children with autism through motor abnormalities [18]. Researchers are increasingly recognizing the link between impaired motor function and cognitive and social aspects in individuals with autism [19]. Motor deficits are prominent in children with autism and may serve as a risk indicator for the early stages of autism [20,21]. Over 67% of children with autism develop motor developmental deficits, resulting in poor motor coordination [22]. The earliest observable symptoms of autism involve motor behaviors, with many children exhibiting rigid, delayed movements and extremely uncoordinated behavior [23]. There was a significant difference in stereotypic behavior between children with autism with and without motor coordination problems [14]. When age, gender, and comorbidities were controlled for, the severity and intensity of stereotypic behavior were higher in children with motor coordination problems than in children without motor coordination problems. Enhancing motor coordination in children with autism not only aids in improving their daily motor skills but also significantly elevates their social interaction and cognitive functions, creating more beneficial conditions for their comprehensive development. This comprehensive intervention not only influences physiological motor abilities, but also provides children with autism with broader and more positive opportunities for social and cognitive development.

It was shown that vitamin A supplementation improves motor incoordination in the valproic-acid-treated rat model of autism by modulating RORα in the cerebellum in vitamin A deficiency [24]. Retinoic acid supplementation ameliorates motor incoordination through the prenatal valproic acid exposure of RARα-CBLN2 in the cerebellum of a rat model of autism [25]. Although research on medication-based approaches to ameliorate motor incoordination in autism has progressed, clinical applications are still pending. The medications are exclusively employed in rat models of autism and have not been administered to children with autism. Beyond medication approaches, intervention training can be utilized to enhance motor coordination in children with autism. The motor system can be trained, and it is an important avenue for improvement in individuals with autism [26]. A variety of physical activity programs including ball games, enjoyable games, and targeted games appear to improve the motor skills of youngsters with autism [27]. Tele-delivered yoga improves motor skills, strength, balance, and flexibility in adolescents with autism. Significant increases in leg strength, flexibility, and dynamic balance between the right and left legs were observed over a 5-week period. These results demonstrate the feasibility and potential effectiveness of yoga in improving physical-activity-related skills in adolescents with autism [28]. Due to the rigid and slow movements and extremely uncoordinated behaviors of children with autism, there is currently less effective motor training for children with autism.

### 1.3. Advantages and Potential Effects of Yoga Intervention

Yoga intervention satisfies the requirements for a concurrent intervention targeting both problem behaviors and motor coordination in children with autism. Yoga, as a gentle aerobic exercise, is better suited for children with autism [29]. Yoga intervention has been successfully applied in the autism group and can effectively intervene in the emotional, social, and sleep aspects of children with autism [30,31,32]. In yoga intervention, on the one hand, breathing exercises can relax the stiff and sluggish limbs of children with autism, thereby helping to relax their emotional state and alleviate emotional issues such as anxiety and depression in children with autism [32]. On the other hand, asana training enables children with autism to vent their bad emotions, improve self-control, reduce irritability, and improve emotional expression [33,34,35]. Yoga can also serve as an effective group activity for enhancing the social interaction skills of children with autism [30]. Yoga may be more effective in enhancing the social interaction skills of children with autism compared to their daily reading, arts and crafts activities, and other tabletop games. Additionally, yoga interventions have demonstrated benefits for sleep quality in children with autism in clinical practice [36]. Owing to these advantages, yoga intervention is progressively emerging as a vital tool for ameliorating symptoms in children with autism.

Yoga practice (combined with standard therapy in institutional care) is anticipated to diminish aggression, antisocial behavior, anxiety, depression, and negative affect in institutionalized youth, concurrently enhancing their cognitive flexibility [37]. Yoga mitigates the adverse impact of behavioral disorders on children and adolescents, consequently reducing tendencies toward aggression and violence [38]. Yoga and mindfulness-based interventions are feasible to reduce aggressive behavior, irritability, lethargy, social withdrawal, and noncompliance [39]. A twelve-week yoga intervention yielded a reduction in social withdrawal among children with autism [11]. Yoga intervention can correct many behavioral outcomes in children with autism, including stereotypical behaviors [40]. Yoga can also improve an individual’s social-emotional skills [41]. Significant improvement in motor abilities was observed in preschool children following a yoga intervention [42]. Yoga intervention has beneficial effects on balance, strength, coordination, and flexibility in children aged 10–12 years and can improve balance and flexibility in healthy children [43]. The pieces of evidence above suggest that yoga may have an effective impact on children with autism in terms of reducing problem behaviors and enhancing motor coordination, which deserves to be studied in depth in the future.

Prior studies into yoga interventions for children with autism have predominantly neglected to assess the sustainability of intervention effects, concentrating solely on their immediate impact [32]. Asanas in yoga interventions are not systematically screened and tailored to align with the specific symptomatology levels observed in children with autism [12].

The overall duration of yoga interventions is excessively prolonged, while the duration of each intervention is overly brief [11,28].

Relative to previous studies, the selection of yoga asanas appropriate for children with autism based on the severity of autism introduces an element of greater relevance and effectiveness. This innovation not only increased the personalization of the yoga practice, but also increased its appeal and sustainability for children with autism. Our study also added a delayed measure to assess the ongoing effectiveness of the yoga intervention. Such improvements have long-term and unique contributions to special school intervention programs and parent training, providing a more creative and practical approach to the field of education and rehabilitation. This targeted and innovative yoga intervention is expected to open new avenues for enhancing problem behaviors and motor coordination in children with autism, providing useful lessons for future research and practice.

## 2. Materials and Methods

### 2.1. Participants

Seventeen children with autism and their teachers were recruited for this study. Inclusion criteria for participants with children with autism: (a) children who had been confirmed to be autistic by psychiatrists or psychologists; (b) children with somatic disorders, epilepsy, respiratory disorders, and other illnesses were excluded; (c) intellectual disability, with some skills significantly delayed and others close to age level; (d) moderate abnormalities in verbal communication: lack of language or meaningful language confused with inappropriate language (imitation speech or inexplicable words); and (e) moderate disorders in body use: signs of moderately specific finger or body posture dysfunction, rocking and spinning, finger wiggling, toe walking. Autism severity was assessed using the Autism Behavior Checklist developed by Krug [44]. Before the experiment, the severity of autism section was filled in by the teacher of the child with autism. During and at the end of the experiment, questionnaires on problem behaviors and test records on motor coordination were filled out by the same teacher of the child with autism.

### 2.2. Measures

#### 2.2.1. Problem Behavior

Teachers used the Aberrant Behavior Checklist (ABC) to assess the participants’ problem behaviors. The ABC has 58 questions in 5 dimensions: Irritability, Social Withdrawal, Stereotypic Behavior, Hyperactivity, and Inappropriate Speech [45]. Each dimension is rated on a 4-point scale. The total scale score is obtained by adding the scores (0 to 3) for each dimension. Higher scores indicate more severe symptoms of problem behaviors.

#### 2.2.2. Motor Coordination

The Movement Assessment Battery for Children—Second Edition (MABC2) was used to assess the motor coordination ability of children with autism [46]. MABC2 has three sets of tests based on three age groups (3–6 years; 7–10 years; and 11–16 years), with the difficulty of the tests deepening or different test programs implemented as the age increases. Although different tests are designed for different age groups, each test set contains the following three motor skills. Manual dexterity: coin throwing, bead threading, and line drawing; ball skills: bean bag catching and bean bag throwing; and static and dynamic balance: one-legged balance, perched-legged walking, and carpet-end hopping. These three main motor abilities are the basic components of all motor coordination abilities that can be fully realized during children’s growth and development. As a standardized test of motor coordination, the MABC2 has been shown to be effective in providing comprehensive information about children’s motor coordination development [47]. The reliability and validity of the MABC2 fulfills the conditions for it to be used as a suitable instrument for assessing children’s motor development [48]. The participants were pretested prior to the formal testing. The results of the pretest showed that the motor coordination of all subjects was only appropriate for the measurement conditions of the MABC2 at the age of 3–6 years. Therefore, the MABC2 at the age of 3–6 years was used as the final measurement tool for the participants’ motor coordination in this study.

### 2.3. Procedure

Using allocation concealment in the randomized controlled trial method, 17 participants were randomly assigned to different groups, including 9 in the experimental group (receiving the intervention) and 8 in the control group (not receiving the intervention). Children with autism in the experimental group underwent yoga intervention (3 sessions/week, 45–50 min/session) in a group for 8 weeks. The yoga intervention took place in the familiar school environment of the child with autism, the intervention process was performed by a specialized principal investigator, and the measured information was recorded by a schoolteacher who was familiar with the child. The intervention protocol is a special yoga protocol for children with autism that focuses on two components: breathing exercises and asana practice [49]. Yoga training asanas are mainly divided into three stages: warm-up, asana strengthening, and relaxation. The first part is warm-up, including cat pose, mountain pose, spine pose, sitting pose, open shoulder pose, and chair pose; the second part is asana strengthening, including downward dog pose, standing pose, and tree pose; the third part is relaxation asana, including stick pose, butterfly pose, child’s pose, seated forward bend pose, seated twisting pose, and lying down pose. After testing and considering the safety issues, the stick pose was deleted. During each group training session, the next asana was only performed when 80% of the children had completed the asana movement. The experimental group had more than 90% attendance. The control group only completed daily program activities and did not participate in yoga training. Children with autism in the control group had a 90% attendance rate at daily activities.

The problem behavior and motor coordination of the participants in the yoga and control groups were measured 4 times. Pre-test: pre-test measurements were taken 1 week before the yoga intervention; mid-test: mid-test measurements were taken after 4 weeks of the yoga intervention; post-test: post-test measurements were taken after 8 weeks of the yoga intervention; delayed test: delayed test measurements were taken 4 weeks after the end of the yoga intervention. Delayed test measurements at 4 weeks after the end of the intervention provided a more comprehensive and holistic understanding of the long-term effects of the yoga intervention on children with autism. This time interval helps to determine whether the effects of yoga are sustained and provides insight into the changes that may occur in children after receiving the yoga intervention.

### 2.4. Data Analysis

The data were analyzed using the software JASP (0.18.0.0) for descriptive statistics, correlation, and a repeated measures ANOVA. The correlation analysis aimed to find out whether there was any significant association between the experimental group and the control group in terms of gender, age, and severity of autism. Repeated measures ANOVA allows for comparing changes at multiple time points, controlling for individual differences, and analyzing trends. In this study, there were multiple time points to control for the effects of the same participants before and after the yoga intervention. Since the participants in this study were all children with autism, there was a great deal of individual variation. Individual differences can be controlled for through a repeated measures ANOVA. The repeated measures ANOVA also allowed for a more complete understanding of how the effects of the yoga intervention evolved over time.

## 3. Results

There were no significant differences in the baseline demographic characteristics of the participants in the two groups in terms of age [*t* (15) = 1.389, *p* = 0.185] and gender [χ^2^ = 0.142, *p* = 0.707]. Autism severity was not significantly different between the experimental group (M = 95.33, SD = 11.15) and the control group (M = 97.63, SD = 10.01), *p* > 0.05, as shown in Table 1.

### 3.1. Problem Behavior

The repeated measures ANOVA showed a significant main effect of the intervention for problem behavior [F_1,15_ = 9.228, *p* < 0.01, η^2^ = 0.356], with a significant problem behavior x group interaction [F_3,45_ = 41.651, *p* < 0.01, η^2^ = 0.029]. The large effect size showed that our yoga intervention was necessary for application to autism problem behaviors. Effect sizes are not dependent on sample size like statistical significance levels. Even when the sample is small, the findings may still be practically significant when the effect size is large. This helps to avoid significance simply because of a larger sample, when in reality there is no significant effect. We further analyzed a two-way interaction between problem behaviors and group to examine between-group differences in problem behaviors (Table 2). After 4 weeks of intervention, the problem behaviors of the children in the experimental group were not significantly lower than those of the children in the control group (Bonferroni adjusted *p* = 0.337, d = 1.381 or large effect). Four weeks may not be enough time to observe significant changes in problem behaviors. Some interventions may take longer to produce significant results. Children’s problem behaviors change over time. After 8 weeks of intervention, the problem behaviors of the children in the experimental group were significantly lower than those of the children in the control group (Bonferroni adjusted *p* = 0.046, d = 1.847 or large effect). One month after the end of the intervention, the problem behaviors of the children in the experimental group were borderline significantly lower than those of the children in the control group (Bonferroni adjusted *p* = 0.054, d = 1.809 or large effect). This showed that the 8-week yoga intervention was effective in reducing the problem behaviors of children with autism. This provides important insights into the development of yoga programs in special schools for two months and beyond.

Changes in the five different dimensions of problem behaviors between the two groups at different times were further analyzed (Table 3). After 4 weeks of intervention, it was found that the irritability and social withdrawal of the children in the experimental group were significantly lower than those of the children in the control group. After 8 weeks of intervention, children in the experimental group were found to have significantly lower irritability and social withdrawal than children in the control group. One month after the end of the intervention, children in the experimental group still had significantly lower irritability and social withdrawal than children in the control group. These results showed that the 8-week yoga intervention is highly effective and has sustained positive effects in reducing problem behaviors in children with autism.

### 3.2. Motor Coordination

The repeated measures ANOVA had a significant main effect on the intervention for motor coordination ability (F_1,15_ = 8.301, *p* < 0.05, η^2^ = 0.298) and a significant motor coordination ability x between-groups interaction (F_3,45_ = 38.912, *p* < 0.01, η^2^ = 0.064). The large effect sizes indicated the relevance of yoga intervention to improve motor coordination in children with autism. We further analyzed the two-way interaction between motor coordination ability and group to investigate between-group differences in motor coordination ability (Table 4). After 8 weeks of intervention, children in the experimental group had significantly higher motor coordination than children in the control group (Bonferroni adjusted *p* = 0.034, d = 1.898, or large effect). One month after the end of the intervention, children in the experimental group still had significantly higher motor coordination than children in the control group (Bonferroni adjusted *p* = 0.027, d = 1.947, or large effect).

Changes in the three different dimensions of motor coordination between the two groups at different times were further analyzed (Table 5). After 4 weeks of intervention, it was found that the ball skills of the children in the yoga intervention group were significantly higher than those of the children in the control group. After 8 weeks of intervention, children in the yoga intervention group were found to have significantly higher ball skills and static and dynamic balance than children in the control group. One month after the end of the intervention, children in the experimental group still had significantly higher ball skills and static and dynamic balance than children in the control group. These results demonstrate the effectiveness and sustainability of the 8-week yoga intervention in improving motor coordination in children with autism.

## 4. Discussion

In order to investigate the effect of yoga intervention on problem behaviors and motor coordination in children with autism, the recruited participants were randomly divided into an intervention group and a control group. The intervention group underwent an 8-week yoga intervention (three times per week for 45–50 min each time), while the control group did not participate in yoga training and only participated in daily program activities. The effect of yoga intervention on the problem behaviors and motor coordination of children with autism was measured four times (pre-test, mid-test, post-test, and after delayed test). The results showed that the yoga intervention was effective in reducing problem behaviors and improving motor coordination in children with autism.

### 4.1. Analysis of the Effectiveness of Yoga Intervention for Problem Behavior in Children with Autism

The problem behaviors of the children with autism in the experimental group were significantly lower than those of the children in the control group after the intervention, as well as after one month. We found that the yoga intervention reduced irritability and social withdrawal in children with autism, thereby reducing problem behaviors. This is consistent with previous research. Yoga training was also found to be beneficial in reducing the irritability and social withdrawal of problem behaviors in children with autism in a 12-week study of a 45 min structured yoga intervention in four special schools [11]. Structured yoga is also able to alleviate symptoms of problematic behaviors in large groups of children with autism [12]. These findings all indicated that yoga intervention can reduce problem behaviors in children with autism. Compared to other studies, our study design shortens the intervention period, which is more conducive to its promotion and application as a course. The results of the delayed test showed that our study resulted in a longer-lasting effect of the yoga intervention.

Yoga exercises have a more positive effect on reducing sympathetic nervous system activity and autonomic balance, resulting in a physiological calming effect [12]. This may help to reduce anxiety levels and alleviate problem behaviors in children with autism. Regular school-aged children showed improved behavior in social interactions after yoga training [50]. During group yoga practice, children with autism have the opportunity to interact with others. This social experience may help to improve social skills and reduce problem behaviors associated with social disorders. A short yoga practice can improve social-emotional behavior in normal preschoolers [51]. Yoga teaches children with autism some self-regulation skills such as deep breathing, meditation, and relaxation techniques. These skills may help children to cope better with mood swings and reduce the occurrence of problem behaviors. This evidence suggests potential mechanisms for yoga intervention for problem behaviors in children with autism.

### 4.2. Analysis of the Effects of Yoga Intervention on Motor Coordination in Children with Autism

Yoga training is effective in improving motor coordination in children with autism. There was a significant improvement in the motor coordination of the children with autism who underwent yoga training as compared to the control group. There are two reasons why yoga may explain the promotion of motor coordination in children with autism. On the one hand, aerobic exercise enhances cortical adaptation to motor skill learning, thereby improving coordination and adaptation of movement [52]. The asana training used in yoga training, such as in the phantom chair pose, downward facing dog pose, and battle II pose, requires the participation of different cerebral cortexes of the child, and through these asanas, the cerebral cortex can be directly stimulated, so yoga training improves the motor coordination of children with autism. Research has also shown that children with autism have cerebellar developmental disorders, and exercise activities can adequately stimulate the cerebellum, thus improving the motor coordination of children with autism [53]. On the other hand, the process of yoga asana training involves a range of motor skills to perform different asanas, which requires the simultaneous involvement of visual, auditory, and proprioceptive senses in children with autism. These sensory systems are considered the basis of sensory integration to develop an individual’s motor coordination. Sensory integration training can improve an individual’s balance by increasing muscle activity in the standing limb muscles and trunk extensors, as well as enhancing stability [54]. The use of different asanas in yoga interventions enhanced the muscle activity of children with autism and thus improved their motor coordination. These two reasons are an important basis for yoga training to enhance motor coordination in children with autism.

We also found that ball skills and static and dynamic balance were most significantly improved in children with autism in the yoga intervention group. Ball skills were significantly higher in the yoga intervention group than in the control group from the middle of the intervention. The completion of the ball skills program required good hand–eye coordination and a high level of concentration. For example, catching a sandbag with both hands required children to coordinate their hand–eye movements and actively adjust their movements according to the direction of the bag. The yoga intervention was found to be effective in improving the subjects’ concentration by increasing the time they spent focusing on a single asana. There was no test of attention in this study, and suitable tests of attention can be added in future studies. At the end of the intervention, children in the yoga intervention group had significantly higher static and dynamic balance than children in the control group. “Vestibular Oriented Sensory Integration Training” is effective in improving balance in children with developmental disorders [55]. Children with autism in the intervention group were fully mobilized visually, auditorily, and proprioceptively during yoga asana training, resulting in improved balance.

### 4.3. Limitations and Future Research

The study has some limitations. ASD is a neurodevelopmental disorder that involves difficulties in social interaction, communication, and behavior. Yoga, as an integrative physical and mental practice, can provide some benefits for some children with autism, but its limitations in this regard need to be recognized. Firstly, ASD shows great individual variation. For some children, yoga may be a beneficial addition to therapy, but for others, it may have limited effect. Each child has their own unique needs and challenges, so different interventions may work differently for different individuals. Secondly, children with autism may be characterized by a lack of concentration, which may make it difficult for them to focus on their yoga practice. Yoga often requires a degree of concentration and self-control, which can be a difficult task for some children with autism. Lastly, children with autism often face challenges with speech and communication, and yoga often emphasizes body language and breathing. Understanding and following the instructions of a yoga instructor can be a challenge for some children, especially if there is a lack of verbal skills.

Despite these limitations, some yoga practices may still be beneficial for some children with autism. When using yoga as part of an intervention for a child with autism, it is important to ensure individualized and specialized instruction to meet the unique needs and ability levels of each child. To provide multiple types of detailed operation videos for yoga training, it is convenient for parents or teachers to conduct yoga training with children with autism, which is one of the individualized ways to integrate yoga into the treatment of children with autism. Using soft music in yoga intervention or utilizing voice guidance from the main test can help children with autism to maintain their attention. Music and sound can be a focus of concentration for children with autism. Shifting attention to body sensations allows children with autism to feel the nuances of each movement. This body sensation focus can help eliminate other distractions. Allowing the teacher, who is familiar with children with autism, to patiently adjust the body language and breathing required for yoga training helps the child with autism to receive the full benefit of yoga intervention training.

## 5. Conclusions

The study conducted an 8-week yoga intervention for children with autism. The results found that the yoga intervention was effective in reducing problem behaviors and improving motor coordination in children with autism. Yoga intervention significantly reduced problem behaviors in children with autism, mainly regarding irritability and social withdrawal. The improvement in motor coordination was observed mainly in ball skills and static and dynamic balance. Yoga is a relatively easy activity to implement, and the results of this study have positive implications for practical applications in a family and school setting. Parents and educators may consider incorporating yoga into the daily activities of children with autism. Future research could compare yoga interventions with other common treatments to understand their relative effectiveness, which could help provide parents and professionals with more comprehensive treatment options.

## Figures and Tables

**Table 1:** Demographic characteristics of participants at baseline.

Group	Age (M ± SD)	Gender (M:F)	Autism Severity (M ± SD)
Experimental Group (*n* = 9)	11.11 ± 2.52	6:3	95.33 ± 11.15
Control Group (*n* = 8)	12.75 ± 2.31	6:2	97.63 ± 10.01
*p*	0.185	0.707	0.664

**Table 2:** Comparison of means and standard deviations of problem behaviors between the two groups at different time points.

	Experimental Group (M ± SD)	Control Group (M ± SD)	Cohen’s d	*p*-Bonf.
Pre-test	87.56 ± 12.13	95.50 ± 7.93	0.816	1.000
Mid-test	82.56 ± 11.52	96.00 ± 7.65	1.381	0.337
Post-test	77.89 ± 10.57	95.88 ± 7.77	1.847	0.046 *
Delayed test	77.89 ± 10.25	95.50 ± 7.96	1.809	0.054 *

Note: * *p* < 0.05 within-group post-to-post comparison.

**Table 3:** Comparison of mean and standard deviation of problem behaviors of different dimensions at different time points between the two groups.

		Irritability	Social Withdrawal	Stereotypic Behavior	Hyperactivity	Inappropriate Speech
Pre-test	Experimental Group (M ± SD)	20 ± 2.55	24.56 ± 5.15	15.44 ± 3.84	21.89 ± 6.15	5.67 ± 1.23
	Control Group (M ± SD)	22.5 ± 4.24	26.63 ± 2.56	16.5 ± 2.83	23.75 ± 4.10	6.13 ± 1.25
	*p*	0.156	0.321	0.533	0.481	0.457
Mid-test	Experimental Group (M ± SD)	18.89 ± 2.52	22.56 ± 5.00	14.56 ± 3.78	20.89 ± 5.84	5.78 ± 1.20
	Control Group (M ± SD)	22.63 ± 4.31	26.75 ± 2.32	16.63 ± 2.62	23.88 ± 4.05	6.00 ± 1.51
	*p*	0.043 *	0.047 *	0.215	0.245	0.740
Post-test	Experimental Group (M ± SD)	17.89 ± 2.42	20.89 ± 4.54	13.78 ± 3.53	19.67 ± 5.20	5.56 ± 1.42
	Control Group (M ± SD)	22.63 ± 4.31	26.63 ± 2.39	16.63 ± 2.62	23.88 ± 4.05	6.25 ± 1.67
	*p*	0.012 *	0.006 **	0.081	0.085	0.369
Delayed test	Experimental Group (M ± SD)	17.22 ± 2.28	21.11 ± 4.26	14.11 ± 3.02	19.44 ± 4.98	6 ± 1.23
	Control Group (M ± SD)	22.5 ± 3.89	26.88 ± 1.96	16.38 ± 2.33	23.25 ± 4.33	6.5 ± 1.20
	*p*	0.003 **	0.003 **	0.107	0.116	0.409

Note: * *p* < 0.05, ** *p* < 0.01 within-group post-to-post comparison.

**Table 4:** Comparison of means and standard deviations of motor coordination between the two groups at different time points.

	Experimental Group (M ± SD)	Control Group (M ± SD)	Cohen’s d	*p*-Bonf.
Pre-test	5.22 ± 2.91	4.25 ± 1.04	0.382	1.000
Mid-test	7.56 ± 3.245	4.38 ± 1.06	1.249	0.567
Post-test	9.33 ± 3.71	4.50 ± 0.93	1.898	0.034 *
Delayed test	9.33 ± 3.43	4.38 ± 1.30	1.947	0.027 *

Note: * *p* < 0.05 within-group post-to-post comparison.

**Table 5:** Comparison of mean and standard deviation of motor coordination of different dimensions at different time points between the two groups.

		Manual Dexterity	Ball Skills	Static and Dynamic Balance
Pre-test	Experimental Group (M ± SD)	3.78 ± 3.27	6.11 ± 1.36	10.22 ± 3.70
	Control Group (M ± SD)	2.38 ± 0.74	5.50 ± 1.93	9.25 ± 2.38
	*p*	1.000	1.000	1.000
Mid-test	Experimental Group (M ± SD)	5.22 ± 4.02	8.78 ± 0.83	12.33 ± 3.08
	Control Group (M ± SD)	2.50 ± 0.54	5.50 ± 1.77	9.38 ± 2.13
	*p*	1.000	0.007 **	1.000
Post-test	Experimental Group (M ± SD)	6.56 ± 5.27	10.67 ± 1.32	14.11 ± 2.32
	Control Group (M ± SD)	2.75 ± 1.04	5.88 ± 1.55	9.13 ± 2.10
	*p*	0.805	<0.001 ***	0.028 *
Delayed test	Experimental Group (M ± SD)	6.44 ± 4.88	10.22 ± 1.20	14.22 ± 2.44
	Control Group (M ± SD)	2.38 ± 0.52	5.63 ± 1.85	9.25 ± 2.19
	*p*	0.498	<0.001 ***	0.028 *

Note: * *p* < 0.05, ** *p* < 0.01, *** *p* < 0.001 within-group post-to-post comparison.

## Data Availability

Data are available on request due to restrictions, e.g., privacy or ethical.
